# Patient-reported recovery in upper abdominal cancer surgery care: A prospective study

**DOI:** 10.1177/00368504211016938

**Published:** 2021-05-12

**Authors:** Anna Johansson, Jessica Karlsson, Victoria Fomichov, Anna Lindhoff Larsson, Per Sandström, Bergthor Björnsson, Jenny Drott

**Affiliations:** 1 Department of Surgery and Department of Clinical and Experimental Medicine, County Council of Östergötland, Linköping University, Linköping, Sweden; 2 Unit for Public Health and Statistics, County Council of Östergötland, Linköping University, Linköping, Sweden; 3 Division of Nursing Science, Department of Health, Medicine and Caring Sciences, Linköping University, Linköping, Sweden

**Keywords:** Surgery, patient-reported measurements, recovery, upper abdominal cancer, supportive cancer care

## Abstract

The study aimed to describe and analyse patient-reported recovery in patients after upper abdominal cancer surgery. This study had a quantitative design and patients were consecutively included in a university hospital in southern Sweden. Twenty-four patients answered the Postoperative Recovery Profile (PRP) questionnaire at three measurement points. All five dimensions were affected. In the physical symptoms dimension, the majority of patients reported a lack of energy upon discharge. High levels of anxiety were reported. Over 50% of patients reported some degree of depressed mood at all three measurement points. In the social dimension, the majority of patients reported some degree of being dependent on help from others in everyday life at 4 weeks after discharge. Few patients are fully recovered at 4 weeks after discharge. Individual patient-reported recovery estimates may be valuable in identifying and planning interventions tailored to each patient’s needs throughout the care process.

## Background

Upper abdominal cancer surgery causes a disability that affects patients in daily life. Many factors influence the recovery process.^1^ In the care of cancer patients who undergo surgery, multimodal concepts (e.g. ERAS and fast track) are increasingly used with extensive approaches.^2^ The aim is to reduce the surgical stress response, reduce the risks of postoperative complications and improve immediate postoperative recovery.^3,4^ Shorter hospital stays include postoperative recovery at home, often with the support of family members.^5,6^ Postoperative recovery differs across individuals. It is important for the patient’s perioperative progression and is seen as a process even after returning home.^1,7^ Postoperative recovery has traditionally been associated with post-surgery physiological recovery, often including observations of vital signs, blood samples and postoperative complications. Studies have shown differences in how patients and staff experienced postoperative recovery.^8,9^

Health care professionals often have a strong focus on clinical improvement, while patients focus on how things will be in the future, how to return to normal life and how to regain their identity.^8^ According to a conceptual analysis by Allvin et al.,^8^ postoperative recovery is described as an energy-intensive process involving four dimensions: physiological, psychological, social and habitual recovery dimensions. The aim of the process is to re-achieve preoperative status.^10^ The complexity and extent of postoperative recovery makes the concept difficult to assess (similar to the concept of health), and experiences of recovery are subjective.^1,11^ The need for support and information during the care of patients with upper abdominal cancer is important^12^ and may affect the patient’s recovery and quality of life.^13^ Multidisciplinary interactions are of paramount importance when cancer patients undergo surgery.^14^ The goal is to achieve the best result for the patient in terms of survival, symptom relief, participation, recovery and well-being.

There is limited knowledge of how patients estimate the actual recovery process at baseline (presurgery/previous trauma) upon discharge from the ward and in their home environment. An increased knowledge of how patients feel and experience their recovery can be of value in being able to work preventively within the multidisciplinary team and can improve the quality and security of the care process. Therefore, patients’ estimation of baseline status and postoperative recovery is of interest to investigate. Current research on postoperative recovery mainly examines colorectal, general and orthopaedic surgery.^1,2,8,9^ To our knowledge, there is a knowledge gap regarding patient-reported recovery among patients who have undergone upper abdominal cancer surgery. Therefore, the focus of this study was to increase the knowledge of this topic with the goal of providing optimal supportive cancer care. This study aimed to describe and analyse patient-reported recovery in patients who underwent surgery for upper abdominal cancer. The research questions were as follows. How do patients grade their recovery over time? Are there any differences between patients’ level of recovery before and after surgery?

## Methods

### Design

The study has a prospective design with a quantitative approach.

### Study participants and data collection

The data collection was carried out by having patients reply to a paper questionnaire about their recovery at three timepoints. The study was carried out in a surgical clinic at a university hospital in southern Sweden between September and December 2019. Patients who met the inclusion criteria were consecutively asked face-to-face to participate during the pre-surgery meeting with physicians (surgeons) and nurses (pre-pandemic/covid-19). The patients were given oral and written information about the study. The inclusion criteria were patients over 18 years of age who underwent elective curative surgery for pancreatic, liver, biliary tract and gallbladder cancer. The patients with liver metastasis were Stage 4. The exclusion criteria were severe cognitive impairment that inhibited patients from completing the questionnaires and patients who did not fluently write or speak Swedish. Thirty-eight patients were included during the study period. Two patients were excluded due to severe complications and ICU care. The timepoints were baseline/preoperative (A), upon discharge from the ward (B) and 4 weeks after discharge from the ward (C). Patients who did not respond to all three timepoints were excluded due to the study design (*n* = 14). Patients who did not respond to the questionnaire were contacted by phone and reminded by the research team. Twenty-four patients answered the questionnaires about their recovery at all three timepoints. The timepoints were baseline/preoperative (A), upon discharge from the ward (B) and 4 weeks after discharge from the ward (C). Twenty-four patients answered the questionnaires about their recovery at all three timepoints. All questionnaires were coded, and data were treated confidentially.

### Measurements

The validated postoperative recovery profile (PRP) questionnaire was used to measure patient-reported postoperative recovery in this study and has previously published elsewhere.^15,16^ The PRP questionnaire has been developed and psychometrically tested on patients, nurses, district nurses and surgeons and previously published elsewhere.^15^ In addition, it has been validated and reliability tested previously in the surgical context on patients who underwent colorectal, orthopaedic, gynaecology and urology surgery.^15,16^ The questionnaire contains five dimensions of recovery: physical symptoms, physical function, psychological, social and activity dimensions.^15^ The author of the questionnaire approved its use in this study. The questionnaire contained 17 and 19 statements/questions. The 17-statement version is adapted for patients in the ward, and the 19 claims are adapted for discharged patients at home. The answer options are structured as a 4-point Likert scale, including severe, moderate, mild or none. Since this is an instrument that measures physical symptoms, physical function, psychological, social and activity dimensions, we decided to also examine patients’ data before surgery (baseline). These patients often receive oncological treatment preoperatively, which may have an impact on patients’ perceived health. Repeated measurements included baseline and postoperative data to gain knowledge about patients’ health status over time.

### Data management and statistical analysis

Descriptive statistics were used to present demographic data and patient estimates from the PRP. Data were analysed at each measurement point (A, B and C), the proportions of patients reporting severe, moderate, mild and no symptoms were calculated. Patients’ degree of recovery was calculated according to a global indicator. A higher value indicated a higher degree of recovery. This global indicator was defined by the number of questions reported as ‘none’ and has been converted into a verbal recovery scale with five degrees of recovery: fully recovered, almost fully recovered, partially recovered, slightly recovered or not at all recovered. The baseline indicator sum was also used to examine the patients’ preoperative status. An analysis with Repeated Measures MANOVA was carried out to examine whether the proportions of each response option changed among the timepoints. All the statements were included in the multivariate analysis except for sexual activity and re-establishing everyday life because they all the data missing for measurement point B. A Repeated Measures ANOVA were carried out instead for these two statements. The response options were dichotomized into a dummy and tested pairwise. The means of the measurements were also compared pairwise among the timepoints with Repeated Measures MANOVA. A Repeated Measure MANCOVA was also performed with three covariates (sex, age and Clavien-Dindo), none of them resulted significant in the analysis. This analysis is not shown. The results of the analysis are presented as point estimation with the margin of error and the post hoc tests *p*-value corrected with Bonferroni. The used significance level was of 5%. Analyses were performed in IBM SPSS Statistics 25.

### Ethical considerations

All patients were given oral and written information about the study. Consent to participate was obtained from all patients, and written informed consent was obtained. The study was performed in accordance with the Declaration of Helsinki and was approved by the Regional Ethical Review Board in Linköping, Sweden (record no: 2016/276-31/2019-04442). Clinical Trials.gov ID: NCT04061902.

## Results

The results are based on 72 completed PRP questionnaires. Twenty-four patients responded to the questionnaire at all three timepoints. The response rate was 63.5% and internal missing were presented in Tables and Figures. The demographic data of the included patients are presented in Table 1. The majority of respondents were men (58.3%). Half of the patients had surgery due to liver metastases, and 50% had postoperative complications. The Clavien-Dindo classification was used for the classification of postoperative complications.^17^ None of the three covariates; sex, age and Clavien-Dindo resulted significant in the analysis.

**Table 1.** Demographic and clinical characteristics of the study population (*n* = 24).

	*n* (%)
Sex	
Male	14 (58, 3)
Female	10 (41, 7)
Age (year)	
Mean	71
Min-max	59–81
SD	7
Cancer diagnosis	
HCC^a^	3 (12, 5)
Liver metastasis	12 (50, 0)
Gallbladder	1 (4, 2)
Pancreatic	8 (33, 3)
Surgery method	
Laparoscopic surgery	1 (4, 2)
Open surgery	23 (95, 8)
Hospital stay (days)	
Mean	8
Min-max	5–28
SD	5
ASA- classification^b^	
ASA 1	2 (8, 3)
ASA 2	15 (62, 5)
ASA 3	7 (29, 2)
ASA 4–ASA 6	0
Complications	
Yes	12 (50, 0)
No	12 (50, 0)
Clavien-Dindo^c^	
Grade 1	12 (50,0)
Grade 2	6 (25, 0)
Grade 3a	3 (12, 5)
Grade 3b	2 (8, 3)
Grade 4a	1 (4, 2)
Grade 4b–Grade 5	0

Normal postoperative process = Grade 1. A higher number indicates more severe complications.

a Hepatocellular carcinoma.

b ASA physical status classification system.^18^

c Classification of complications.

### Global level of recovery and graded recovery over time

The global level of recovery was calculated at each measurement point and is shown in Table 2. At baseline, one patient was classified as fully recovered. At 4 weeks after discharge, none of the patients had fully recovered. Over 60% of patients were not fully recovered at measuring points B and C (Table 2).

**Table 2.** Global level of recovery according to the PRP.

Recovery	Measurement point A	Measurement point B	Measurement point C
	Baseline, preoperative (*n* = 24)	Discharge (*n* = 24)	4 weeks after discharge (*n* = 24)
	*n* (%)	*n* (%)	*n* (%)
Fully recovered	1 (4.2)	0 (0.0)	0 (0.0)
Almost fully recovered	8 (33.3)	0 (0.0)	2 (8.3)
Partially recovered	10 (41.2)	5 (20.8)	5 (20.8)
Slightly recovered	1 (4.2)	3 (12.5)	2 (8.3)
Not at all recovered	4 (16.7)	16 (66.7)	15 (62.5)

### Patient-reported recovery over time

**Figure 1.**
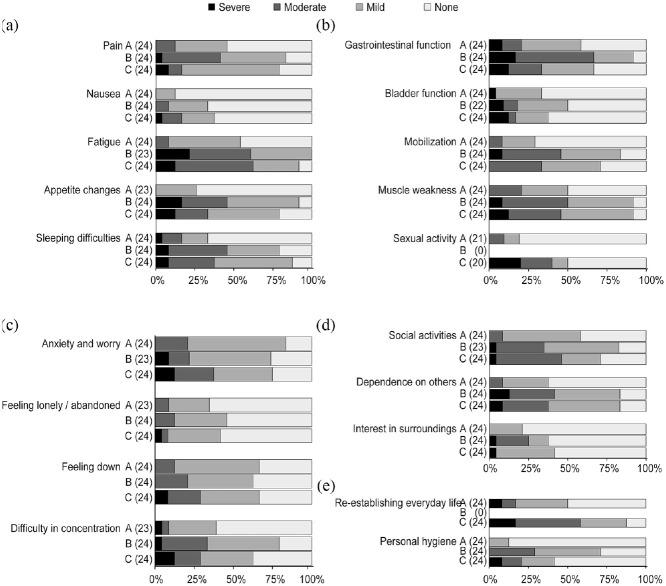
(a) Estimation of the physical symptom dimension according to the PRP, (b) estimation of the physical function dimension according to the PRP, (c) estimation of the psychological function dimension according to the PRP, (d) estimation of the social function dimension according to the PRP and (e) estimation of the activity dimension according to the PRP.

Figure 1(a) to (e) describe the dimensions of physical symptoms, physical function, psychological, social and activity at each measuring point (A, B and C). In the physical symptoms dimension (Figure 1(a)), all patients reported a lack of energy upon discharge from the ward (one missing answer). Over 75% of patients reported some degree of pain, an impact on appetite and an impact on sleep at measuring points B and C.

In the physical function dimension (Figure 1(b)), the majority of respondents reported varying degrees of muscle weakness upon discharge from the ward and 4 weeks after discharge. At measuring point B, 50% of patients reported a moderate effect on gastrointestinal function. The questionnaire for measurement point B did not assess sex life, so estimates were only measured at measurement points A and C (three and four patients did not answer the question). High levels of anxiety were reported. Over 50% of patients reported some degree of depressed mood at all three measurement points (Figure 1(c)).

In the social dimension, as shown in Figure 1(d), over 80% of patients reported some degree of inconvenience with respect to being dependent on help from others upon discharge from the ward and at 4 weeks after discharge.

In the activity dimension, over 85% of patients had some degree of restriction in their everyday life at 4 weeks after discharge. The questionnaire at measuring point B did not assess restrictions in everyday life, which meant that measurements were carried out only at measurement points A and C (Figure 1(e)).

### Differences between patients’ recovery at baseline, upon discharge from the ward and 4 weeks after discharge

Among the patients who reported no preoperative symptoms on the included dimensions, there were statistically significant differences in several dimensions between measurement points A and C. Nausea, fatigue, appetite changes and sleeping difficulties were significantly different between these measuring points *p* < .05 (data not shown). Mobilization, muscle weakness, dependence on others and re-establishing everyday life were also significantly different (*p* < .05) between baseline and 4 weeks after discharge (data not shown). Table 3 illustrates the estimated mean of symptoms. Significant differences were found in terms of a nausea, lack of energy, appetite, sleep over time. Between measurement points B and C, there was no significant difference in any of the dimensions, except for pain. Estimated percentage of moderate/severe symptoms over time for each measuring point, including baseline (A), discharge from the ward (B) and 4 weeks after discharge (C), are presented in Table 4.

**Table 3.** Differences in estimated mean of recovery over time (*n* = 24).

Time	Estimated mean (MOE)*			*p*-value**		
	A	B	C	A-B	A-C	B-C
Physical symptoms						
Pain	1.5 (±0.4)	2.4 (±0.4)	1.9 (±0.3)	0.004	0.164	0.045
Nausea	1.0 (±0.0)	1.3 (±0.3)	1.5 (±0.4)	0.167	0.045	0.647
Fatigue	1.6 (±0.3)	2.8 (±0.4)	2.6 (±0.4)	0.000	0.001	1.000
Appetite changes	1.2 (±0.2)	2.5 (±0.5)	2.2 (±0.4)	0.000	0.001	0.896
Sleeping difficulties	1.4 (±0.4)	2.4 (±0.4)	2.4 (±0.4)	0.016	0.001	1.000
Physical functions						
Gastrointestinal function	1.8 (±0.5)	2.6 (±0.5)	2.1 (±0.5)	0.020	1.000	0.110
Bladder function	1.4 (±0.4)	1.7 (±0.5)	1.6 (±0.5)	0.490	0.617	1.000
Mobilization	1.4 (±0.4)	2.2 (±0.5)	2.0 (±0.4)	0.010	0.021	0.647
Muscle weakness	1.7 (±0.4)	2.4 (±0.4)	2.5 (±0.4)	0.002	0.002	1.000
Sexual activity***	1.3 (±0.3)		2.1 (±0.6)		0.009	
Psychological						
Anxiety and worry	1.9 (±0.3)	2.1 (±0.5)	2.2 (±0.5)	0.781	0.866	1.000
Feeling lonely/abandoned	1.4 (±0.4)	1.6 (±0.4)	1.5 (±0.4)	1.000	1.000	0.997
Feeling down	1.7 (±0.4)	1.8 (±0.4)	1.9 (±0.5)	1.000	0.647	0.997
Difficulty in concentration	1.5 (±0.4)	2.1 (±0.4)	1.9 (±0.5)	0.040	0.330	1.000
Social						
Social activities	1.6 (±0.3)	2.1 (±0.3)	2.1 (±0.4)	0.002	0.103	1.000
Dependence on others	1.4 (±0.3)	2.4 (±0.4)	2.2 (±0.4)	0.001	0.015	0.781
Interest in surroundings	1.2 (±0.2)	1.6 (±0.5)	1.4 (±0.3)	0.269	0.311	1.000
Activity						
Re-establishing everyday life***	1.8 (±0.4)		2.6 (±0.4)		0.001	
Personal hygiene	1.1 (±0.2)	1.9 (±0.4)	1.6 (±0.4)	0.001	0.047	0.248

* Mean with margin of error at 95% confidence interval estimated with repeated measures MANOVA. Dimensions are coded as: 1 = none, 2 = mild, 3 = moderate, 4 = severe.

** Repeated measures MANOVA pairwise comparisons with Bonferroni correction.

*** Excluded from MANOVA due to missing data at B, analysed with repeated measures ANOVA.

**Table 4.** Estimated percentage of moderate/severe symptoms over time (*n* = 24).

Time	Estimated % of moderate/severe (MOE)*			*p*-value**		
	A	B	C	A-B	A-C	B-C
Physical symptoms						
Pain	12 (±17)	47 (±27)	12 (±17)	0.164	1.000	0.164
Nausea	0 (±0)	6 (±13)	18 (±20)	0.997	0.248	0.490
Fatigue	6 (±13)	59 (±26)	59 (±26)	0.002	0.002	1.000
Appetite changes	0 (±0)	41 (±26)	35 (±25)	0.012	0.028	1.000
Sleeping difficulties	12 (±17)	41 (±26)	35 (±25)	0.288	0.124	1.000
Physical functions						
Gastrointestinal function	18 (±20)	59 (±26)	29 (±24)	0.043	1.000	0.167
Bladder function	6 (±13)	18 (±20)	18 (±20)	0.490	0.490	1.000
Mobilization	12 (±17)	41 (±26)	29 (±24)	0.288	0.563	1.000
Muscle weakness	24 (±23)	41 (±26)	47 (±27)	0.563	0.124	1.000
Sexual activity***	11 (±15)		37 (±24)		0.056	
Psychological						
Anxiety and worry	12 (±17)	24 (±23)	29 (±24)	0.997	0.563	1.000
Feeling lonely/abandoned	12 (±17)	18 (±20)	6 (±13)	1.000	0.997	0.49
Feeling down	12 (±17)	24 (±23)	29 (±24)	0.490	0.248	1.000
Difficulty in concentration	6 (±13)	29 (±24)	24 (±23)	0.124	0.248	1.000
Social						
Social activities	6 (±13)	24 (±23)	41 (±26)	0.248	0.028	0.808
Dependence on others	6 (±13)	41 (±26)	29 (±24)	0.028	0.124	1.000
Interest in surroundings	0 (±0)	24 (±23)	0 (±0)	0.124		0.124
Activity						
Re-establishing everyday life***	17 (±16)		58 (±21)		0.002	
Personal hygiene	0 (±0)	24 (±23)	18 (±20)	0.124	0.248	1.000

* Percentage with margin of error at 95% confidence interval estimated with repeated measures MANOVA.

** Repeated measures MANOVA pairwise comparisons with Bonferroni correction.

*** Excluded from MANOVA due to missing data at B, analysed with repeated measures ANOVA.

## Discussion

The results showed the psychological, physiological, social and habitual dimensions of recovery in this patient population. None of the patients were fully recovered at 4 weeks after discharge according to the global recovery indicator from the PRP. A few (four patients) were not at all recovered at the baseline. These patients often receive oncological treatment preoperatively, which may have an impact on perceived health and status before surgery.^19^ The included patients in this study had many symptoms prior to surgery. Physical symptoms/functions and psychological symptoms were present and may be related to both previous treatments and the disease itself. A diagnosis of cancer often affects the emotional life of both the affected person and their loved ones. In this study, 50% of patients had surgery due to liver metastases, which means that the primary tumour has or will need to be treated, thus impacting the patient’s physical status. The physical deterioration due to cancer and surgical treatment can lead to increased supportive care needs. Physical decline in older patients with abdominal cancer may affect the postoperative phase. The patients in this study had an average age of 71 years, and the results of Karlsson et al.^20^ regarding physical decline may be applicable in this cohort. The results from their study show the importance of improving functional and physical capacity prior to surgery to reduce postoperative physical decline.^20^

Increased preoperative body mass index, decreased preoperative serum albumin, age and skin closure using staples can be a risk factors for wound infection who affect the recovery.^21^

Preoperative anxiety was common, and most of the patients expressed some degree of concern at all three measurement points. More than 50% of patients reported having a depressed mood at all measurement points. These results are consistent with previous research indicating that cancer has been shown to cause psychological symptoms for both patients and the next of kin.^22,23^ Preoperative anxiety was also found in previous studies in which patients were unable to discuss their suffering and healthcare professionals did not identify or confirm these feelings. These psychological factors are difficult to manage and require much effort.^24^ Anxiety can use much energy and can also worsen the experience of pain.

Emotional reactions for example, worry, anxiety and sadness also exist in family members^25^ and may also affect the situation for the patient. Psychological factors such as anxiety and fear may lead to negative preoperative attitudes towards the surgical outcome, which have been shown to have adverse effects on most factors of postoperative recovery and may contribute to an extended recovery process.^22–24^

Anxiety and fears should be identified and addressed early. Psychosocial support, information and good communication are important both during the investigation period and before and during curative treatment.^26^ Adequate and appropriate information has been shown to contribute to security, such that patients feel more prepared for surgery and can take more responsibility for their own care.^27^ A lack of information can lead to insecurity, anxiety and a worse recovery.^24,27^ In this study, 50% of patients had varying degrees of postoperative complications. Complications have been shown to prolong the length of care and complicate the recovery process.^24,28^

Over 80% of the patients reported being inconvenienced by being dependent on other people to cope with their daily lives and reported restrictions in their daily lives. These results are consistent with previous research showing that patients often wish to regain independence in physical activities as soon as possible to regain autonomy.^29^

One-third of the included patients in this study had pancreatic cancer. It is worth noting that studies of patients with pancreatic cancer are often difficult to implement due to the disease severity and fast progression. However, the severity, progression and poor survival may require interventions that are both quick and effective. A previous longitudinal study showed a burden of unmet needs in this patient population. Early attention to pain and anxiety may reduce unmet supportive care needs.^13^ Optimizing supportive management is important for improving quality of life and recovery.^30,31^

By providing holistic and person-centred care pre- and postoperative surgical care, patient health may be improved. Individualized optimization strategies, such as prehabilitation programmes, will focus on different stressors already in the preoperative phase. Effective symptom monitoring and engaging patients who are already in the preoperative phase may provide an option to affect recovery and reduce postoperative deconditioning.^32–34^

The instrument used in the study is psychometrically used to measure postoperative recovery. The choice of instruments related to validity and reliability is a strength. The results of the study are based on individual estimates of 24 patients, and generalizability is transferable in similar surgical context. However, a larger sample size had increased the probability of generalizability in terms of the population. The results of this study contribute important knowledge regarding the care process and may provide a basis for future interventions. To work more with a person-centred approach, it is important to quickly identify symptoms and identify functions that are affected. The multidisciplinary team needs to work with the patient preoperatively, during the time in the ward prior to discharge, and after discharge via follow-up and return visits.

### Clinical implications

Short hospital stays after surgery and the fact that a large part of the recovery takes place in the patient’s home environment present a challenge for patients, next of kin and healthcare professionals. The results of this study on recovery may contribute to the knowledge of the multidisciplinary team involved in pre- and postoperative treatment and care for patients with upper abdominal cancer. How patients rate the recovery process over time can be helpful in designing effective strategies, patient information and individual interventions. Therefore, the measurement of patient recovery may be a part of the standard clinical routine in the future. By providing holistic and person-centred care, patient health may be improved.

### Study limitations

The PRP questionnaire covers several dimensions of recovery and gives a picture of how the individual rated recovery but does not provide a deeper understanding of the cause of the estimates. The sample size may have affected the results and the interpretation of the results. In the study, there were missing answers in the questionnaires. Internal missing may contribute to variance and a risk of misleading estimates. Most internal missing in the study concerned the items which included an estimate of ‘impact on sex life’. Sexual life was one of those items that was less relevant during the construction of the questionnaire and was therefore removed at the measurement point with 17 items adapted for the ward. In this study, three patients in measuring point A and four in measuring point C did not choose to answer the question, which is interpret by the research team an indication that it is a sensitive issue.

## Conclusion

Patients undergoing surgery for upper abdominal cancer are affected by psychological, physiological, social and habitual factors, with most estimates of these symptoms increasing after surgery according to the PRP. Few patients are fully recovered at 4 weeks after discharge, and the majority of patients experience symptoms preoperatively. Individual patient-reported recovery estimates based on preoperative status and follow-up data may be valuable in identifying and planning interventions tailored to each patient’s needs throughout the care process.
